# Management of COVID-19 in Kidney Transplant Recipients: A Single-Center Case Series

**DOI:** 10.1155/2022/9636624

**Published:** 2022-08-18

**Authors:** Maruhum Bonar H. Marbun, Riahdo J. Saragih, Tantika Andina

**Affiliations:** Division of Nephrology and Hypertension, Department of Internal Medicine, Cipto Mangunkusumo General Hospital, Jakarta, Indonesia

## Abstract

**Background:**

Kidney transplant recipients (KTRs) were reported to be at higher risk of developing severe coronavirus disease-2019 (COVID-19). Despite being one of the most impacted countries, little is known about KTRs with COVID-19 in Indonesia. This report aims to explore the management strategies and short-term clinical outcomes of KTRs with COVID-19 in an Indonesian transplant center.

**Methods:**

We observed KTRs who were admitted following COVID-19 diagnosis. Anamnesis, physical, laboratory, and radiologic examinations were performed. Demographic and transplant histories were recorded, along with symptoms, vaccination status, and management related to COVID-19.

**Results:**

Nineteen KTRs were observed and 14 (73.6%) were male. The most common presenting symptoms were fever, cough, and shortness of breath. Nine (47.3%) KTRs had severe-critical COVID-19. The mortality rate was 42.1%. Acute kidney injury (AKI) was present in six (31.6%) of KTRs, five (83.3%) of whom were nonsurvivors. The median D-dimer level was higher in nonsurvivors (5,800 versus 670 *μ*L), while other laboratory parameters were comparable. Seven (36.8%) KTRs were vaccinated. The mortality rates of vaccinated and unvaccinated KTRs were 14.2% and 70%, respectively. Antiviral therapy, anticoagulant, intravenous immunoglobulin, and tocilizumab were prescribed to 89.5%, 89.5%, 15.8%, and 10.5%, respectively. Immunosuppressive therapy (IST) was halted in 68% of KTRs, among which 61.5% passed away.

**Conclusion:**

The clinical presentation of COVID-19 in KTRs was similar to that in the general population, whereas the mortality rate was higher. Management strategies for KTRs with COVID-19 should include prevention of AKI and hypercoagulation. Vaccination seems to be beneficial for KTRs, while temporary withdrawal of IST does not.

## 1. Introduction

Coronavirus disease-2019 (COVID-19) pandemic caused by the infection of severe acute respiratory syndrome coronavirus disease (SARS-CoV-2) has brought immense challenges worldwide. COVID-19 manifests variably, ranging from asymptomatic infection to critical illness requiring mechanical ventilation. Kidney transplant recipients (KTRs) were reported to be at higher risk of developing more severe illness. KTRs have impaired immune systems due to the prolonged use of immunosuppressive therapy (IST), which renders them vulnerable to infectious diseases. The majority of KTRs have comorbidities such as hypertension, diabetes mellitus, and cardiovascular diseases, which are widely reported as predictors of COVID-19 mortality [1]. Therefore, KTRs with COVID-19 require specific management strategies because the treatment of COVID-19 may interfere with their ongoing chronic treatment. In addition, COVID-19 often causes acute kidney injury (AKI), particularly to those who have underlying kidney damage, which may worsen the prognosis of KTRs with COVID-19. To date, there have been no standardized guidelines for managing KTRs with COVID-19. Thus, the management strategies of KTRs with COVID-19 in many centers were investigational.

Indonesia is one of the most affected Asia Pacific countries, struggling with limited healthcare and testing facilities [2]. Despite being one of the most impacted countries, little is known about KTRs with COVID-19 in Indonesia. While previous case reports have documented the clinical characteristics of KTRs with COVID-19 in several developed countries, the findings may not reflect the situation in Indonesia and other developing countries as we struggle with limited healthcare and testing facilities. This case series explores the management strategies and short-term clinical outcomes of KTRs with COVID-19 in an Indonesian transplant center.

## 2. Methods

We included all adult KTRs who received kidney grafts at any time, with a COVID-19 diagnosis confirmed by positive polymerase chain reaction (PCR) tests on nasopharyngeal and oropharyngeal swabs between March 2020 and September 2021. We recorded the baseline characteristics of the patients, which include demographics and clinical characteristics related to the transplant history. We also recorded clinical presentation, hospital stay, management strategies, and short-term outcomes of the patients.

Obesity was defined as having a body mass index (BMI) > 30 kg/m^2^ at the time of admission. Patients were regarded as vaccinated if they had received two doses of COVID-19 vaccination prior to admission. Laboratory testing was performed on the day of admission in the Clinical Pathology Laboratory of Cipto Mangunkusumo Hospital (CMH). D-dimer level was measured using a Sysmex 5100 analyzer (Siemens Healthcare Diagnostics, Marburg, Germany). C-reactive protein (CRP) and interleukin-6 (IL-6) levels were measured using a Cobas analyzer (Roche Diagnostics, GmbH, Mannheim, Germany). Verbal consent was obtained from the participants before drawing blood samples.

## 3. Results

### 3.1. Baseline Characteristics

A total of 21 patients who received kidney grafts in our center contracted COVID-19 in the period between March 2020 and September 2021, among whom two received outpatient treatment and therefore were excluded from the series. 19 KTRs were hospitalized following COVID-19 diagnosis. 14 (73.6%) patients were male. The median age of the patients was 46.7 years. Only Patient 1 and Patient 16 were older than 60 years at the time of diagnosis. All patients received kidney grafts from living donors. Posttransplant time ranged from 9 months to 8 years, with an average of 3.89 years. Only Patient 4 underwent kidney transplant less than one year prior to COVID-19 diagnosis. At presentation, all patients were on routine IST, comprised by a combination of calcineurin inhibitors and antimetabolite drugs. All patients were prescribed tacrolimus. Mycophenolate mofetil/mycophenolic acid and azathioprine were prescribed to 17 (89.4%) and 2 (10.5%) patients, respectively. Everolimus was prescribed only to patient 14, who previously experienced adverse effects of other immunosuppressive drugs. Steroid was prescribed to 15 (78.9%) KTRs.

Various comorbidities were present in 15 (78.9%) patients, with hypertension and diabetes mellitus being the most commonly found. Coronary artery disease was present in four (21.1%) recipients. Patient 13 was on a permanent pacemaker. Five (26.3%) patients were obese. Patients' baseline characteristics are summarized in Table 1.

### 3.2. COVID-19 Infections

The average time from symptom onset to hospital admission was 4.20 days. Mild, moderate, and severe-critical COVID-19 were present in six (31.6%), five (26.3%), and nine (47.3%) patients, respectively. The most common presenting symptoms were fever and cough; both were present in 17 (89.5%) patients. Shortness of breath was reported by 10 (52.6%) patients: eight (42.1%) patients had severe-critical illness, one (5.26%) patient had moderate disease, and one (5.26%) had mild disease. Gastrointestinal symptoms were present in five (26.3%) of the participants. Laboratory tests were performed on the day of admission. The median white blood cell (WBC) and lymphocyte count were 6.59 (1.92–30.56) x1000/*μ*L and 18.35 (1.60–149), respectively. The median serum creatinine level at admission was 1.8 (0.6–8.5) mg/dL. During hospitalization, six (31.6%) patients had ≥0.3 mg/dL elevation of serum creatinine level, indicating the presence of acute kidney injury (AKI). Three (15.8%) patients underwent renal replacement therapy (RRT). Interleukin-6 level was obtained from nine (47.3%) patients, among which three (15.8%) patients had IL-6 level of more than 40 ng/mL. 18 (94.7%) patients showed bilateral infiltrates on the thorax X-ray, and only one (5.26%) patient showed no acute changes.  Table 2 summarizes COVID-19 infections and management in our patients.

At a median follow-up of 12 days, 11 (57.9%) patients were discharged home, while eight patients passed away, giving a mortality rate of 42.1%. Patient 5, a 34-year-old male with no comorbidities other than CKD, became the only patient who developed severe-critical COVID-19 and survived. The rest of nonsurvivors had at least one comorbidity other than chronic kidney disease.

We attempted to compare the clinical characteristics of survivors and nonsurvivors. Nonsurvivors tend to be older (51 versus 47 years) and underwent kidney transplants more recently (4.5 versus 3.5 years) compared to survivors. While survivors and nonsurvivors reported similar COVID-19 symptoms, shortness of breath was more frequently reported in nonsurvivors. In contrast, gastrointestinal symptoms were only found in survivors. The median C-reactive protein (CRP) (95 versus 36 mg/L) and D-dimer (5,800 versus 670 *μ*L) levels were higher in nonsurvivors, while other laboratory parameters between the two groups were comparable. Six (31.6%) patients developed AKI during hospitalization, five (83.3%) of whom did not survive. All patients who underwent RRT during hospitalization did not survive.

Vaccination status was known in 17 (89.5%) patients, among which seven (41.2%) were vaccinated, while 10 (58.8%) were not. Among those who were vaccinated, six (85.7%) patients survived, including a severe-critical patient. The median age of vaccinated survivors was 55 years (39.25–55.75). The median time after transplant of vaccinated survivors was 4.5 (3.25–5.75). Meanwhile, among those who were unvaccinated, seven (70%) passed away and only three (30%) survived. Hence, the proportion of nonsurvivors was markedly higher in unvaccinated patients (70% versus 14.2%). The median age of unvaccinated nonsurvivors was 51 (33.5–54.5). The median time after transplant of unvaccinated nonsurvivors was 4 (2.5–4.5). Furthermore, among nine patients who developed severe-critical illness, the only survivor was vaccinated. Patient 11 was the only one who had received COVID-19 vaccination and did not survive after 26 days of hospitalization.

### 3.3. Management of COVID-19

All patients were admitted to COVID-19 isolation ward. Seven (36.8%) patients required mechanical ventilation and therefore were admitted to the Intensive Care Unit (ICU). Pharmacologic therapy consisted of antivirals, anticoagulants, intravenous immunoglobulin, and tocilizumab. Antivirals were prescribed to 17 (89.5%) patients. Patients who presented with moderate and severe symptoms at presentation were prescribed remdesivir, while those with mild symptoms were prescribed favipiravir or oseltamivir. Five (26.3%) patients were initially given favipiravir before later switching to remdesivir due to deteriorating conditions. Patient 18 was given lopinavir/ritonavir before switching to remdesivir. 17 (89.5%) patients received anticoagulant therapy. Intravenous immunoglobulin was prescribed to three (15.8%) patients, while tocilizumab was prescribed to two (10.5%) patients, all of whom had severe-critical COVID-19.

The IST regimen was unchanged in six (31.5%) patients, among which four (66.7%) had mild COVID-19. IST regimen was changed in only one severe-critical COVID-19 patient who later survived. In contrast, IST was halted in 13 (68.4%) patients, consisting of eight severe-critical COVID-19 patients, four moderate COVID-19 patients, and one patient with mild disease. In the said patients, both antimetabolite drugs and calcineurin inhibitors were withdrawn temporarily at least during hospitalization. Among those patients, eight (61.5%) were nonsurvivors.

## 4. Discussion

In our case series, we discussed the clinical characteristics, management strategies, and short-term outcomes of 19 KTRs with COVID-19 admitted to our center. The demographic characteristics of our participants are comparable to those reported in other case series [3]. Similar to those reported in other case series, our patients have a high frequency of comorbidities, with hypertension and diabetes mellitus being the most common. Around 47% of recipients had severe-critical illness, possibly signifying the extent of concern when transplant recipients did contract the infection.

The most common presenting symptoms in KTRs with COVID-19 in our center were fever, cough, and shortness of breath. The presenting symptoms appear to be similar to a report on the general, nonimmunosuppressed population in Jakarta, Indonesia, in which most patients present with multiple symptoms of cough, fever, malaise, and shortness of breath [4]. The number of patients reporting gastrointestinal symptoms was slightly higher than that reported in the general population. Higher prevalence of gastrointestinal symptoms in KTRs may reflect recipients' vulnerability to coinfection by intestinal parasites and cytomegalovirus [5].

The median length of hospital stay in our case series is longer compared to the previous case series by Alberici et al. [6], in which the median length of inpatient stay was seven days. Patient 11 was hospitalized for 26 days, the longest out of all our patients. Although initially admitted due to COVID-19 infection, the patient was tested negative on follow-up PCR, 14 days after admission. Therefore, it was presumed that inflammatory processes persist even after the viral infection subsides, leading to delayed resolution, worsening symptoms, and prolonged hospital stay.

The mortality rate in our patient series was notably higher than that reported in the general population, which ranged from 2.3 to 12%, possibly reflecting the importance of underlying health conditions in overcoming the infection [4]. The mortality rate of KTRs with COVID-19 in the other centers ranged from 25% to 30% [1]. Higher mortality rates in our center may be associated with delayed hospitalization. In our report, the average time from symptom onset to hospital admission was 4.20 days. Alberici et al. [6] suggested that COVID-19 patients who had previously received kidney grafts require an aggressive management strategy and thus may benefit from early hospitalization.

In our patient series, the median CRP (95 versus 36 mg/L) level was higher in nonsurvivors. Several previous studies have reported CRP as a predictor of COVID-19 severity and mortality. CRP is an acute phase protein which quickly elevates during infection or inflammation. CRP production by hepatocytes is mainly stimulated by cytokines including IL-6. Cytokine storm, characterized by massive release of IL-6 and other cytokines, frequently developed in severe COVID-19, leading to a marked elevation of CRP [7].

### 4.1. AKI in KTRs with COVID-19

AKI was widely known as an important COVID-19 complication in the general population, as well as a predictor of disease severity and mortality in COVID-19 patients. AKI is particularly prevalent in COVID-19 patients with underlying kidney damage. AKI related to SARS-CoV-2 infection may be mediated by intravascular volume depletion; ischemia, acute tubular necrosis (ATN); direct nephron injury associated with medication; and ischemia associated with a hypercoagulable state. The pathological state of the kidney is probably caused by complement activation and cytokine release. SARS-CoV-2 is known to bind angiotensin-converting enzyme 2 receptors, which are expressed in the kidney. Thus, it is possible that AKI in COVID-19 patients resulted from direct destruction of kidney cells [1]. These mechanisms may also occur in kidney grafts, worsening renal function during COVID-19 infection. Therefore, it is essential to include strategies to prevent AKI in the management of KTRs with COVID-19.

In our patient series, AKI was present in six patients admitted to our center, among which only one patient survived. The incidence was higher compared to that reported in the general population [4]. This emphasizes the importance of AKI management when treating KTRs with COVID-19. Meanwhile, in another report of KTRs with COVID-19 in Iran, AKI was present in all patients in the cohort. Other case series reported various proportions of KTRs with COVID-19 requiring renal replacement therapy, ranging from 27% to 57% [3,8]. Therefore, it is important for physicians to ensure optimal strategies to prevent AKI in KTRs with COVID-19. Two of the patients who developed AKI required a vasopressor during hospitalization, indicating prerenal factors underlying AKI development. While it is common to restrict fluid in patients in respiratory distress to avoid overload, fever and gastrointestinal loss due to COVID-19 infection often cause relative volume depletion. Thus, COVID-19 patients may require intermittent monitoring of volume status to prevent hypovolemia and prerenal causes of AKI [9,10]. Meanwhile, four of the patients who developed AKI did not show any signs of intravascular volume depletion. The high D-dimer levels in said patients may suggest that ischemia associated with hypercoagulable state was responsible for AKI development.

### 4.2. Management of COVID-19

Similar to other case series, our center also uses antiviral therapy to treat KTRs with COVID-19. To date, there have been no approved antiviral drug for the treatment of COVID-19 in KTRs, hence the investigational use in many centers [11]. In our center, antiviral therapy was prescribed in accordance with the recommendation of COVID-19 management in Indonesia. In the more recent versions of the guidelines, favipiravir and remdesivir are recommended for therapy of moderate and severe COVID-19 patients. A previous report by Ardhi et al. concluded that remdesivir is more suitable for COVID-19 patients, particularly those with severe disease [12]. Patients with severe COVID-19 who received remdesivir tend to demonstrate better outcomes as well as shorter time to recovery. Meanwhile, a previous report on the effectiveness of favipiravir for treating KTRs with COVID-19 concluded that favipiravir does not appear to affect mortality [13]. Therefore, several patients in our center who were initially on favipiravir were later prescribed remdesivir after showing deteriorating conditions.

Anticoagulant is prescribed in the majority of our patients. COVID-19 infection is widely known to cause coagulopathy, which results from an inflammatory endothelial cascade and hypoxia, resulting in a hypercoagulable state commonly manifested as D-dimer elevation [14]. In addition, kidney transplant itself is associated with a chronic hypercoagulable state, particularly during the first six months, as it is considered influenced by several immunosuppressive agents, original nephropathy, pretransplant dialysis modality, and secondary polycythaemia [15]. Accordingly, our participants also showed elevated D-dimer. Similarly, an elevated D-dimer in KTRs with COVID-19 was also reported in another case series by Alberici et al. [6]. Therefore, KTRs are prone to hypercoagulability and anticoagulation is required in KTRs with COVID-19. Early anticoagulation is associated with lower AKI incidence in KTRs with COVID-19, as it prevents hypercoagulation and thrombotic microangiopathy.

Only two patients in our cohort received tocilizumab. Tocilizumab, a humanized antibody against interleukin-6 (IL-6) receptor, is believed to be able to assist in managing cytokine storm in COVID-19 patients. Trujillo et al. suggested that treatment with tocilizumab may be associated with improved clinical outcomes in KTRs with COVID-19 [16,17].

As we followed the recommendation of Kidney Disease: improving Global Outcomes (KDIGO), patients in our cohort received a combination of immunosuppressive drugs including calcineurin inhibitor (CNI) and antimetabolite drugs (AD). KDIGO also recommends adjusting the dosage of immunosuppressive therapy in case of viral infection [17]. However, to date, there have been no standardized guidelines regarding the management of IST in KTRs with COVID-19.

IST was a halt in all our patients with severe-critical illness, except for Patient 5. Concomitantly, Patient 5 was the only patient who developed severe-critical illness and survived. It was commonly presumed that temporary withdrawal and/or dose adjustment of immunosuppressive therapy may assist in optimizing the host immune response essential for battling a viral infection. Despite the presumed beneficial effects, it is important to note that severe symptoms in KTRs were linked to a disproportionate release of inflammatory cytokines [1]. The majority of nonsurvivors had negative PCR results at the time of outcome, indicating that the inflammation process persists even after the viral infection subsides. Several reports propose that KTRs with COVID-19 may actually benefit from their immunosuppressed conditions. Thus, it is possible that continuing immunosuppressive therapy in KTRs with COVID-19 exerts beneficial effects on survival.

Nevertheless, it is important to note that Patient 5 was the only patient with severe-critical illness who did not have any comorbidities other than CKD. Therefore, it is possible that the better outcome of Patient 5 is not merely due to the maintenance of immunosuppressive therapy but also because of the better baseline underlying health condition. It signifies the importance of comorbidities in affecting the severity and outcomes of COVID-19. A previous study has suggested that multiple comorbidities are associated with severe symptoms, disease complications, and progression to critical disease [18].

### 4.3. COVID-19 Vaccination

Our findings support the previous study which suggested the benefit of COVID-19 vaccination in KTRs. Although the safety and efficacy of COVID-19 vaccination in solid organ transplant recipients were not clearly known as most vaccine trials did not include transplant recipients, available data suggest that COVID-19 vaccines are safe to use in chronic kidney disease patients [19]. In our patient series, the proportion of nonsurvivors was markedly higher in unvaccinated patients. Furthermore, the only patient who had severe-critical illness and survived was Patient 5, who had received a second dose of COVID-19 vaccination prior to infection, thus emphasizing the importance of vaccination in KTRs. Nevertheless, healthcare personnel may have different approaches to decide whether a patient with an underlying medical condition was eligible to receive vaccination. The fact that Patient 5 was eligible to receive COVID-19 vaccine may reflect the adequate baseline underlying health condition prior to infection.

It is also important to note that one patient (Patient 11) received COVID-19 vaccination and still did not survive. It might be explained by findings from previous studies which suggested that COVID-19 deaths after complete vaccination were associated with poor antibody response to the vaccine. Recent studies also described suboptimal antibody response by KTRs to SARS-CoV-2 vaccines [20–22]. Decreased humoral response was associated with older age, treatment with mycophenolic acid (MPA), dialysis duration before transplantation, and allograft dysfunction, as well as low lymphocyte count at the time of vaccination [20, 21]. In addition, better humoral response was demonstrated by KTRs who received SARS-CoV-2 prior to transplantation [22].

Similarly, in our patient series, Patient 11 was older than the median age of vaccinated survivors. Patient 11 also had longer time after transplant, which may reflect longer exposure to MPA treatment, compared to the median. Furthermore, Patient 11 was older than the median age of the unvaccinated nonsurvivors, although the times after transplant were similar. This finding emphasizes better benefit of COVID-19 vaccination in younger KTRs.

## 5. Conclusions

In this report, the presentation of COVID-19 in KTRs is similar to that in the general population, whereas the mortality rate was higher. Management of COVID-19 in KTRs should include strategies to prevent AKI and hypercoagulation, since both seem to be associated with mortality. Based on our experience in managing COVID-19 in KTRs, it appears that temporary withdrawal of IST was not beneficial for survival. Meanwhile, early vaccination seems to be beneficial in reducing the risk of developing severe-critical illness as well as mortality, particularly in younger KTRs and those who have shorter exposure to MPA. However, this conclusion could not be made due to the limited sample size.

## Figures and Tables

**Table 1:** Baseline characteristics of KTRs with COVID-19.

Patient	Gender	Age (years)	Comorbidities	Time from transplant (years)	IST regimen
1	Male	66	Obesity, DM	4	Tacrolimus, MMF/MPA, steroid
2	Female	31	None	5	Tacrolimus, MMF/MPA, steroid
3	Male	51	DM, hypertension, CAD	3	Tacrolimus, MMF/MPA, steroid
4	Male	58	DM, hypertension, CAD	0.8	Tacrolimus, MMF/MPA, steroid
5	Male	34	None	4	Tacrolimus, MMF/MPA, steroid
6	Female	31	Hypertension	1	Tacrolimus, MMF/MPA, steroid
7	Male	55	DM, hypertension,	3	Tacrolimus, MMF/MPA, steroid
8	Male	30	Hypertension	4	Tacrolimus, MMF/MPA, steroid
9	Female	42	Obesity	8	Tacrolimus, azathioprine, steroid
10	Male	48	Hypertension	2	Tacrolimus, MMF/MPA, steroid
11	Male	58	Hypertension, CAD	4	Tacrolimus, MMF/MPA, steroid
12	Female	33	None	5	Tacrolimus, azathioprine, steroid
13	Male	51	DM, hypertension, CAD, bradycardia with permanent pacemaker, pulmonary tuberculosis, COPD	2	Tacrolimus, MMF/MPA, steroid
14	Male	36	None	5	Tacrolimus, everolimus
15	Male	55	Obesity, hyperuricemia	6	Tacrolimus, MMF/MPA
16	Male	60	Hypertension	6	Tacrolimus, MMF/MPA
17	Male	46	Obesity	6	Tacrolimus, MMF/MPA
18	Female	56	Obesity, DM	5	Tacrolimus, MMF/MPA
19	Male	52	DM, hypertension	3	Tacrolimus, MMF/MPA

CAD: coronary artery disease; DM: diabetes mellitus; IST: immunosuppressive therapy.

**Table 2:** COVID-19 infections and management in KTRs with COVID-19.

Patient	Severity	Symptoms at presentation	AKI	Management	Vaccination status	Outcome
1	Severe-critical	Fever, cough, SOB, fatigue	Yes	Remdesivir, anticoagulant, IVIG, MV, halt IST	Unvaccinated	Nonsurvivor
2	Severe-critical	Fever, cough, SOB	Yes	Favipiravir, anticoagulant, MV halt IST	Unvaccinated	Nonsurvivor
3	Severe-critical	Fever, cough, fatigue	No	Remdesivir, anticoagulant, tocilizumab, MV, halt IST	Unvaccinated	Nonsurvivor
4	Severe-critical	Fever, cough, SOB	No	Remdesivir, anticoagulant, tocilizumab, MV halt IST, RRT	Unvaccinated	Nonsurvivor
5	Severe-critical	Fever, cough, SOB	No	Remdesivir, anticoagulant	Vaccinated	Survivor
6	Mild	Fever	No	Oseltamivir, anticoagulant	Vaccinated	Survivor
7	Mild	Cough	No	Remdesivir	Vaccinated	Survivor
8	Severe-critical	Fever, cough, SOB	Yes	Favipiravir then remdesivir, anticoagulant, IVIG, MV, halt IST, RRT	Unvaccinated	Nonsurvivor
9	Moderate	Cough, SOB, dyspepsia	Yes	Remdesivir, anticoagulant, halt IST	Unknown	Survivor
10	Moderate	Fever, cough, dyspepsia	No	Favipiravir then remdesivir, anticoagulant, halt IST	Unvaccinated	Survivor
11	Severe-critical	Fever, cough	No	Favipiravir then remdesivir, anticoagulant, MV, halt IST	Vaccinated	Nonsurvivor
12	Mild	Fever, cough, SOB, dyspepsia	No	No antiviral, anticoagulant	Unvaccinated	Survivor
13	Severe-critical	Fever	Yes	Chloroquine phosphate, MV, halt IST, RRT	Unvaccinated	Nonsurvivor
14	Severe-critical	Fever, cough, SOB, diarrhea	Yes	Favipiravir then remdesivir, anticoagulant, halt IST	Unvaccinated	Nonsurvivor
15	Mild	Fever, cough	No	Remdesivir, anticoagulant	Vaccinated	Survivor
16	Moderate	Fever, cough, dyspepsia	No	Remdesivir, anticoagulant, halt IST	Vaccinated	Survivor
17	Mild	Fever, cough	No	Favipiravir then remdesivir, anticoagulant, halt IST	Unknown	Survivor
18	Moderate	Fever, cough	No	Lopinavir/ritonavir then remdesivir, anticoagulant, IVIG	Vaccinated	Survivor
19	Moderate	Fever, cough, diarrhea	No	Remdesivir, anticoagulant, halt IST	Unvaccinated	Survivor

AKI: acute kidney injury; IVIG: intravenous immunoglobulin; IST: immunosuppressive therapy; MV: mechanical ventilation; RRT: renal replacement therapy; SOB: shortness of breath.

## Data Availability

The dataset is available from the Dryad repository (DOI: https://doi.org/10.5061/dryad.g79cnp5rc).
